# Relationship between Mental Disorders, Smoking or Alcoholism and Benign Prostate Disease

**DOI:** 10.3390/clinpract14010020

**Published:** 2024-02-05

**Authors:** Paloma Chantada-Tirado, Venancio Chantada-Abal, José-David Cózar-Ortiz, Cristina Chantada-Tirado, José-Manuel Cózar-Olmo, Manuel Esteban-Fuertes, Andrea Alvarez-Ossorio-Rodal, Javier Flores-Fraile, Magaly-Teresa Márquez-Sánchez, Bárbara-Yolanda Padilla-Fernández, María-Fernanda Lorenzo-Gómez

**Affiliations:** 1 Department of Surgery, University of Salamanca, 37007 Salamanca, Spainmagalymarquez@usal.es (M.-T.M.-S.); mflorenzo@saludcastillayleon.es (M.-F.L.-G.); 2 Urology Department, University Hospital Complex of A Coruña, 15006 A Coruña, Spain; 3 Psychiatry Department, Gómez Ulla Defense Central University Hospital, 28047 Madrid, Spain; 4 Urology Section, Virgen de las Nieves University Hospital, 18014 Granada, Spain; 5 Urology Service, National Hospital for Paraplegics of Toledo, 45004 Toledo, Spain; 6 Primary Care, 41013 Sevilla, Spain; 7 Urology Section, Department of Surgery, University of La Laguna, 38200 Tenerife, Spain; 8 Urology Service, University Hospital of Salamanca, 37007 Salamanca, Spain

**Keywords:** mental disorders, smoking, alcoholism, prostatitis

## Abstract

Introduction: Mental disorders, smoking, or alcoholism and benign prostate disease are highly prevalent in men. Aims: To identify the relationship between mental disorders, smoking, or alcoholism and benign prostate disease. Methodology: A prospective multicenter study that evaluated prostate health status in 558 men from the community. Groups: GP—men who request a prostate health examination and whose medical history includes a mental disorder, smoking, or alcoholism prior to a diagnosis of benign prostate disease; GU—men who request a prostate health examination and whose medical history includes a benign prostate disease prior to a diagnosis of mental disorder, smoking, or alcoholism. Variables: age, body mass index (BMI), prostate specific antigen (PSA), follow-up of the mental disorder, smoking or alcoholism, time elapsed between urological diagnosis and the mental disorder, smoking or alcoholism diagnosis, status of the urological disease (cured or not cured), concomitant diseases, surgical history, and concomitant treatments. Descriptive statistics, Student’s *t*-test, Chi2, multivariate analysis. Results: There were no mental disorders, smoking, or alcoholism in 51.97% of men. Anxiety, smoking, major depressive disorder, pathological insomnia, psychosis, and alcoholism were identified in 19.71%, 13.26%, 5.73%, 4.30%, 2.87%, and 2.15% of individuals, respectively. Nonbacterial prostatitis (31.54%), urinary tract infection (other than prostatitis, 24.37%), prostatic intraepithelial neoplasia (13.98%), and prostatodynia (1.43%) were prostate diseases. Unresolved symptomatic benign prostate disease was associated with anxiety, depression, and psychosis (*p* = 0.002). Smoking was the disorder that men managed to eliminate most frequently. The dominant disorder in patients with symptomatic benign prostatic disease was alcoholism (*p* = 0.006). Conclusions: Unresolved symptomatic benign prostatic disease is associated with anxiety, depression, and psychosis. Alcoholism is associated with a worse prognosis in the follow-up of symptomatic benign prostatic disease.

## 1. Introduction

An association between anxiety, panic disorder, and benign prostate disease has been suggested [1]. The relationship between anxiety, depression, and lower urinary tract symptoms (LUTS) has been researched since at least 1964 [2].

Depression and prostate disease may share common pathophysiological mechanisms such as the potential presence of a psychological trigger, impaired hypothalamic–pituitary–adrenal axis, inflammation, neuroendocrine cascades, sensitization of the central nervous system (CNS) (especially in relation to painful sensations), multifactorial modulation of the CNS, involvement of oxidative stress, and the effects of antidepressants [3].

Smoking may be related to lower prostate volume in men with benign prostatic hyperplasia (BPH) [4].

One hypothesis suggests that, if there is a relationship between mental health and prostatic symptoms, current treatments for these urological disorders may not fully resolve urinary problems if the underlying mental disorders, smoking, or alcoholism are not themselves fully resolved [5].

The lifetime prevalence of anxiety disorders (AD) in the general population ranges from 9.2% to 28.7% worldwide [1,2]; however, there is a lack of information on the relationship between benign urological diseases and mental disorders, smoking, or alcoholism regarding the possible influence on prostate health.

Mental health could affect the appropriate treatment of patients with LUTS, and further in-depth studies are warranted on the possible relationship between mental disorders, smoking, or alcoholism and the response to treatment in patients with LUTS [6].

This study emerged as a substudy investigation within a broader cohort of 2240 men from the community. These individuals had previously taken part in a prostate health research study designed for the public, with the primary objective of identifying a diagnostic marker for prostate cancer (CH10 Code RTC-2017-6271-1, RETOS), due to the high volume of participants who spontaneously attended the consultation and were diagnosed with benign urological diseases and investigated for anxiety or depression. The authors decided to explore the relationship between these pathologies due to their innovative findings and to open a new field of research.

The aim was to identify mental disorders, smoking, or alcoholism, and benign urological diseases in men who request a prostate health exam without suspicion of malignancy, and to establish the relationship between mental disorders, smoking, or alcoholism, and benign urological diseases.

## 2. Methods

A multicenter prospective observational study of 558 men from the community who requested a prostate health exam was conducted from 29 January 2019 to 1 September 2020. The men who spontaneously requested the prostatic health status exam during the recruitment of the study were men aged 49 years and older.

In the Salamanca and Ávila healthcare areas, men from the community over 49 years of age were offered participation in a study to assess their prostate health. This offer was made over mass media (newspaper and radio) as well as directly in the consulting rooms of the primary care health centers where the researchers of the Renal Urological Multidisciplinary Research Group (GRUMUR) of the Salamanca Biomedical Research Institute (IBSAL) work.

The present study emerged as a substudy investigation within a broader cohort of 2240 men from the community. These individuals had previously taken part in a prostate health research study designed for the public based on identifying a diagnostic marker for prostate cancer (CH10 Code RTC-2017-6271-1, RETOS). The sample of men was recruited through spontaneous consultations by men from the community, which has proven to be a suitable and valuable resource for the purposes of this specific investigation.

Patients were recruited in a similar way in both groups: in both groups, the reason for spontaneous consultation by men from the community was to determine their prostate health status. Hence, they are comparable, and the selection is valid. Authors do not start with two different groups of patients with different established diagnoses. It is based on a general sample of the general population’s recruitment due to information in newspapers, radio, or primary care consultations [7]. Therefore, it is considered that the groups are comparable.

The methodology used is considered appropriate for the selection of comparable individuals because the men who have consulted have done so under equal conditions, that is, a desire to investigate their prostate health without suspicion of suffering from prostate cancer.

Prostatitis, prostatic hyperplasia, and LUTS were diagnosed based on the patient’s urinary discomfort, medical history (characteristics and duration of the symptoms of the genitourinary system, previous surgical interventions, especially those that may affect the genitourinary system, general health aspects, including sexual function and concomitant medication), physical examination, rectal examination, and complementary studies (uroanalysis, renal function, and PSA) [8].

Mental disorders, smoking, or alcoholism were diagnosed in the psychiatric consultation based on the criteria of psychiatric specialists and the ICD-11 [9].

The frequency and duration of smoking were defined by the cigarette/tobacco user classification system by the World Health Organization (WHO) [10]. Alcohol consumption was defined through the Standard Drinking Unit (SBU); one SBU was equivalent to 10 g of alcohol. Alcoholism was considered to be 4 SBU for men, according to the WHO [11].

For the BPH questionnaire, the International Prostate Symptom Scale (IPSS) version from the Spanish Association of Urology was used [12], and the quality of life questionnaire was used for anxiety and depression [13].

Inclusion criteria: males over 49 years of age who requested a prostate health exam, who were able to give informed consent, and who did not have a prior diagnosis of prostate disease.

Exclusion criteria: males under 49 years of age, men who had a previous diagnosis of prostate cancer, and men who could not give informed consent to participate in the study.

### 2.1. Study Groups

Two study groups were defined:

GP (psychiatric): men who request a prostate health examination and whose medical history includes a mental disorder, smoking, or alcoholism prior to a diagnosis of prostate disease.

GU (urological): men who request a prostate health examination and whose medical history includes a prostate disease prior to a diagnosis of mental disorder, smoking, or alcoholism.

### 2.2. Variables Studied

Age, body mass index (BMI), prostate specific antigen (PSA), free PSA/total PSA index, PSA velocity in ng/mL per year, follow-up of the mental disorder, smoking, or alcoholism in months, time elapsed between urological diagnosis and the mental disorder, smoking or alcoholism diagnosis, status of the urological disorder (cured or not cured), primary urological diagnosis, secondary urological diagnoses, primary mental disorder, smoking or alcoholism diagnosis, secondary mental disorder, smoking or alcoholism diagnoses, urinary symptoms, concomitant diseases, surgical history, and concomitant treatments.

Primary diagnosis is defined as the diagnosis that appears first in time, that is, the first diagnosis made and that implies in which group the patient is included, whether it is a mental disorder, smoking or alcoholism, or a benign urological disease.

Secondary diagnosis is defined as a disorder that appears during the follow-up of the individual throughout the study, whether it is a mental disorder, smoking or alcoholism, or a benign urological disease, and that can occur in either of the two groups.

### 2.3. Statistical Analysis

The results were analyzed with descriptive statistics, Student’s *t*-test, Chi2, Fisher’s exact test, ANOVA/analysis of variance (with Scheffe’s test for normal samples and Kruskal–Wallis for other distributions), Pearson and Spearman correlation studies, and multivariate analysis. Correspondence analysis, which analyzes the relationships between categories, rows, and columns, and logistic regression were used for the multivariate analysis. The association patterns between the row and column variables are described with symmetric normalization. The inertia absorbed by two factorial axes was 95% (0.9551). This means that the correspondence factor analysis model was appropriate, i.e., a powerful statistical analysis for assessing the general sample and the groups (*p* = 0.0170). Logistic regression shows the relationship between independent and dependent variables. The analysis was performed on the NSSS2006/GESS2007 automatic statistical calculator. Statistical significance was accepted for *p* < 0.05.

### 2.4. Ethical Concerns

The CAAV/2019/22 study protocol was approved by the Clinical Research Committee of the Ávila University Assistance Complex. All collaborating practitioners work in accordance with the current legislation in their respective countries, under public or private health care systems. All participating individuals signed an informed consent form before being included in the study. Clinical information was handled under Directive 2001/20/EC of the European Parliament and Council and in compliance with the Standards of Good Clinical Practice of the Ministry of Health and Consumer Affairs and the Spanish Agency of Medicines.

### 2.5. Costs

Funding for the study was supported by the Renal Urological Multidisciplinary Research Group (GRUMUR) of the Institute of Biomedical Research of Salamanca (IBSAL), 37007 Salamanca, Spain.

## 3. Results

The mean follow-up time of the primary mental disorders, smoking, or alcoholism for all individuals was 93.43 months, SD 78.62, median 89.85, range 1–74 months; the mean time was lower in GU (Mann–Whitney U test, *p* = 0.0001) (Appendix A, Table A1).

The mean follow-up time of the primary urological diseases for all individuals was 40.37 months, SD 42.9, median 18.05, range 0.10–243.98 months; the mean time was lower in GP (Mann–Whitney U test, *p* = 0.0001) (Appendix A, Table A1).

The mean time between the primary urological disease and the primary mental disorder, smoking, or alcoholism in the general sample was 75.52 months, SD 74.49, median 65.51, range from 1 to 726.45 months; the mean time was lower in GU (Mann–Whitney U test, *p* = 0.0025) (Appendix A, Table A1).

### 3.1. Status of Urological Disorder: Cured or Not Cured

All men who participated in this study were diagnosed with a primary urological disorder, but not all were diagnosed with a primary mental disorder, smoking, or alcoholism. Therefore, patients with a primary urological disease had a primary disorder that had already been cured or had not been cured. In the general sample, 380 men (68.1%) had a primary urological disease and were cured, while 178 men (31.9%) had a primary urological disease but were not cured. No difference was found in the distribution of cured urological diseases (Fisher’s exact test, *p* = 0.3670).

### 3.2. Primary Urological Disease

Patients with prostate cancer were intentionally excluded because these patients could have a significant psychological burden. Only patients with benign prostate disease were included.

**Figure 1:**
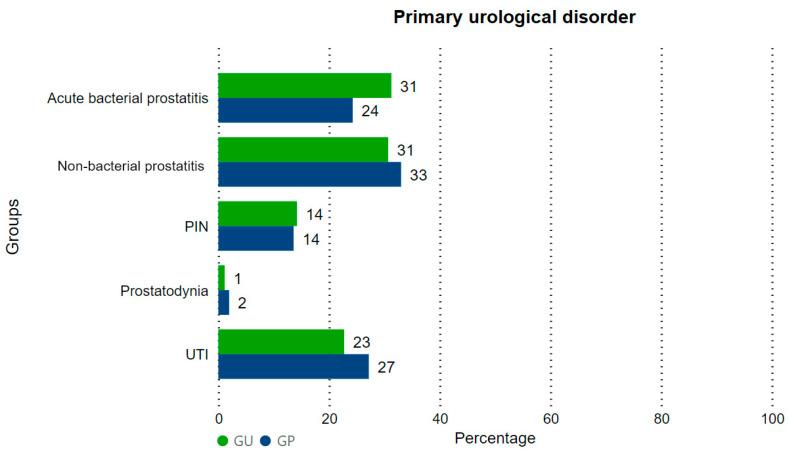
Distribution of the primary urological diseases in GP and GU. PIN: prostatic intraepithelial neoplasia. UTI: urinary tract infection.

In the general sample, 176 individuals (31.5%) had non-bacterial prostatitis, 160 individuals (8.67%) had acute bacterial prostatitis, 136 individuals (24.37%) had a non-prostatitis urinary tract infection, 78 individuals had (13.97%) PIN, and 8 individuals (1.43%) had prostatodynia (Figure 1). There was no difference in the distribution of the main urological diseases (Fisher’s exact test, *p* = 0.3910).

### 3.3. Secondary Urological Diseases

The term “secondary urological diseases” means urological diseases that appear during follow-up. In the general sample, 268 individuals (48%) did not develop secondary urological diseases, while the remainder did develop one or more. These included the following: 202 cases (36.2%) of benign prostatic hyperplasia; 2 cases (0.4%) of nocturia; 8 cases (1.4%) of prostatism; 8 cases (1.4%) of cystitis; 36 cases (6.5%) of urinary tract infection (no prostatitis, no cystitis); 8 cases (1.4%) of bacterial prostatitis; 12 cases (2.2%) of chronic prostatitis; 4 cases (0.7%) of urinary incontinence; 6 cases (1.1%) of prostatodynia; and 4 cases (0.7%) of candidal balanitis. There were no differences between groups in the development of secondary urological diseases (Fisher’s exact test, *p* = 0.081).

### 3.4. Status of Mental Disorders, Smoking, or Alcoholism: Cured or Not Cured

**Figure 2:**
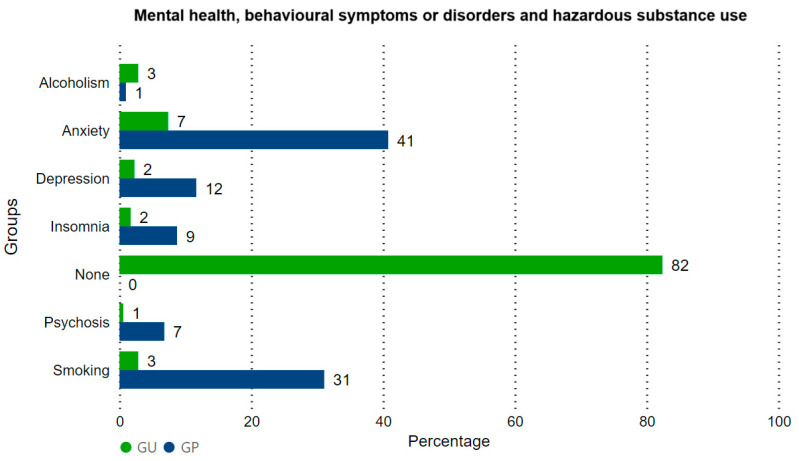
Distribution of the status of the primary mental disorder, smoking, or alcoholism in GP and GU. Positive, not cured: men with a disorder that persisted throughout follow-up. Positive, cured: men with a disorder that was cured during follow-up. Negative: men without mental disorders, smoking, or alcoholism.

In the general sample, 290 men (52%) were not diagnosed with any mental disorder, smoking, or alcoholism. Among those who had any mental disorder, smoking, or alcoholism (48%), 82 men (14.7%) were cured, while 186 men (33.3%) were not cured (Figure 2). A higher percentage of individuals with no mental disorder, smoking, or alcoholism was found in GU; more individuals were diagnosed, either cured or not, in GP (Fisher’s exact test, *p* = 0.007).

### 3.5. Primary Mental Disorders, Smoking, or Alcoholism

In the general sample, 290 men did not have mental disorders, smoking, or alcoholism. There were 110 cases (19.7%) of anxiety, 32 cases (5.7%) of major depressive disorder, 24 cases of insomnia, 74 cases (13.3%) of smoking, 12 cases (3.84%) of alcoholism, and 16 cases of major psychotic disorder. In GU, the absence of mental disorders, smoking, or alcoholism was most frequent (Fisher’s exact test, *p* = 0.0002).

### 3.6. Secondary Mental Disorders, Smoking, or Alcoholism

In the general sample, 6 individuals (1.1%) developed anxiety, 4 individuals (0.7%) developed major depressive disorder, 10 individuals (1.8%) developed insomnia, 39 individuals (7%) became smokers, 17 individuals became alcoholics (3%), 2 individuals (0.4%) had major psychosis, and no disorders were found in 86% of individuals. In GU, individuals most frequently developed anxiety, major psychotic disorder, or did not develop any mental disorder, smoking, or alcoholism (Fisher’s exact test, *p* = 0.00028).

### 3.7. Urinary Symptoms

In this paper, “urinary symptom” refers to symptoms that led to a specific consultation throughout the follow-up; 56 cases were found in the general sample. The following symptoms occurred: pain in 10 individuals (17.9%), haematuria in 8 individuals (14.3%), and other voiding disorders (dysuria, urine stream abnormality) in 38 individuals (67.9%). Consultation for pain (1.94%) and haematuria (2.91%) was more frequent in the GP group, while in the GU group, consultations for other voiding disorders were more frequent (7.95%) (Fisher’s exact test, *p* = 0.031).

### 3.8. General Concomitant Diseases

In the GP group, hypertension, acute myocardial infarction, metabolic disorders, type 2 diabetes mellitus, irritable colon, respiratory disorders, peripheral neurological disorders, arthritis, osteoarthritis, and otorhinolaryngological disorders were more frequent (*p* = 0.0001). In the GU group, dyslipidaemia, trauma disorders, rheumatological disorders, disorders of the central nervous system, ophthalmological disorders, allergies, and the absence of any secondary general disorder were more frequent (Fisher’s exact test, *p* = 0.0001) (Table 1).

### 3.9. Concomitant Treatments

More ACE inhibitors, metformin and other antidiabetics, 5-ARI inhibitors, ranitidine, proton pump inhibitors, benzodiazepines and other psychiatric treatments and second-level analgesics were used in GP (*p* = 0.0001), while in GU there was more use of ARA II, lipid-lowering agents, first-level analgesics, acetylsalicylic acid, various other drugs, or no treatment (Fisher’s exact test, *p* = 0.0001).

### 3.10. Multivariate Analysis

#### 3.10.1. Correspondence Analysis

The principal urological disorders found were prostatitis, acute bacterial prostatitis, urinary tract infections other than prostatitis, prostatic epithelial neoplasia, and prostatodynia.

**Figure 3:**
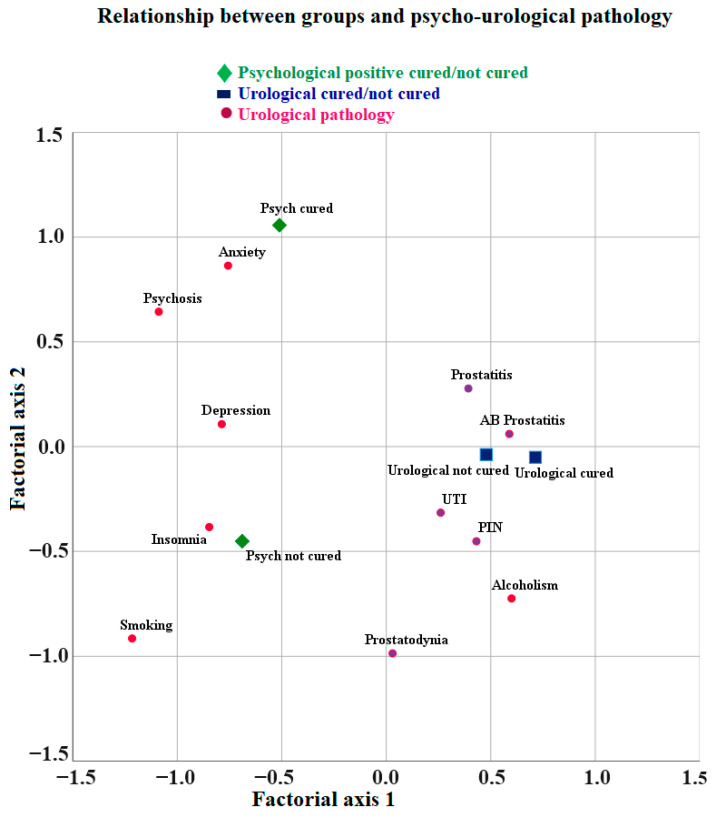
Relationship between groups and mental disorders, smoking, or alcoholism and cured and not cured urological disorders.

Patients with mental disorders, smoking, or alcoholism, both cured and uncured, have more prostatitis and UTIs. Patients with urological diseases that were not cured had acute bacterial prostatitis. Patients with urological diseases that were cured were those with PIN and UTI (urinary tract infection other than prostatitis) (Figure 3).

The most frequent uncured condition was smoking, followed by anxiety, depression, insomnia, and psychosis. The most frequent condition where the patients also have a urological disease, cured or not, was alcoholism. The most frequent conditions in patients without urological disorders were anxiety, depression, insomnia, smoking, and psychosis, with smoking having the highest penetrance (Figure 3).

Patients with uncured mental disorders, smoking, or alcoholism and with uncured urological disorders have higher levels of anxiety, depression, and psychosis (Figure 3).

The most frequently cured mental disorder was anxiety, followed by psychosis. The mental disorders that most frequently were not cured were insomnia and depression, with smoking being the most common. Alcoholism was the most frequent hazardous substance used in the case of both cured and uncured urinary disorders. The urological disorders that were cured were PIN and sporadic UTI, while prostatitis was not cured (Appendix A, Table A2, Figure 3).

#### 3.10.2. Logistic Regression

Results of the logistic regression analyses conducted to identify associations between urological diseases, mental disorders, smoking, or alcoholism with comorbid conditions and risk factors.

Decreasing age (*p* ≤ 0.050), other benign prostatic conditions (*p* = 0.018), other voiding disorders (*p* = 0.042), central and nervous system disorders (*p* = 0.032) and anxiety (*p* = 0.0012) were associated with urological diseases that disappear. Increasing cardiac rhythm disturbances (*p* = 0.025) and depression (*p* = 0.034) were associated with urological diseases that persist.

Among mental disorders, smoking, or alcoholism, decreasing anxiety (*p* = 0.0014), depressive disorder (*p* = 0.033), cardiac rhythm disturbances (*p* = 0.007), metabolic disorder (*p* = 0.019), respiratory disorder (*p* = 0.001), general pain (*p* = 0.005), and osteoarthritis (*p* = 0.042) were associated with resolved mental health disorders. Increased time between mental disorder, smoking or alcoholism diagnosis, and urological diagnosis (*p* = 0.059) was associated with no resolved mental disorders.

## 4. Discussion

This study aims to demonstrate a relationship between mental disorders, smoking or alcoholism, and benign prostate diseases in men from the community over the age of 49 who request a prostate health check without suspicion of malignancy.

One very interesting finding in our study was that 36.91% of the individuals who requested a prostate health exam had some history of mental disorders, smoking, or alcoholism at the time of the first consultation.

The following percentage of mental disorders, smoking, or alcoholism was present in the general sample, from highest to lowest frequency: anxiety (19.71%), smoking (13.26%), major depressive disorder (5.73%), pathological insomnia (4.30%), psychosis (2.86%), and alcoholism (2.15%).

Marital status, occupation, income, education level, hypertension, diabetes, previous medication history, and surgical history, according to the literature, are also influencing factors. Sedentary and chili eating habits may also be important risk factors for prostate diseases. These variables were analyzed, with non-significant findings (*p* => 0.050) in our study. Due to the large number of variables analyzed, only those that were statistically significant (*p* ≤ 0.050) have been described in the article.

### 4.1. Anxiety and Depression

Ahn et al. found that 19.71% of the men who consulted for a prostate health exam had a prior diagnosis of anxiety; the association of anxiety with the benign prostatic disease remains unclear. Stress, accompanied by anxiety, has been suggested as a significant factor in the development, prolongation, and perpetuation of prostate symptoms [14]. Compared to this study, increasing cardiac rhythm disturbances and depression were significantly associated with no cured urological disorders.

Koh JS et al. studied anxiety, depressive disorder, somatization, and LUTS and found that these may be related to the major neurotransmitters 5-hydroxytryptamine, serotonin, and norepinephrine, and psychiatric symptoms could play a role in the development of clinical symptoms and treatment outcomes in patients with LUTS [15,16]. Depression may be a risk factor for prostate disease (the risk is seven times higher) and somatization (the risk is three times higher) compared to men without depression or somatization [17].

In this study, depressive disorder and anxiety may be risk factors for unresolved prostate disease (the risk is three times higher) compared to men with resolved prostate disease. Coyne KS et al. found that men with mixed urinary incontinence have more clinically relevant anxiety (42.1%) than men with other types of UI [18].

Our study has identified an association between symptomatic, uncured benign prostate disease and a higher incidence of anxiety, depression, and psychosis. In addition, patients with symptomatic benign prostate disease are more likely to show anxiety and psychosis during follow-up than patients without prostate disease.

Numerous studies suggest an association between LUTS, anxiety, and/or depression [19,20], although some have reported conflicting results [21]. In addition, LUTS, anxiety, and depression have been reported to have an additive effect on physical and mental wellbeing, as well as quality of life [22]. However, most of these studies rely on self-reported surveys using rating scales for anxiety/depressive disorder rather than on diagnoses confirmed by a physician [23]. Our study is novel in that it is based on rigorous diagnoses issued by physicians.

### 4.2. Smoking and Alcoholism

In the present study, smoking was found to have the best prognosis in terms of cure or disappearance of the disorder, compared to both the primary mental disorders or alcoholism or benign symptomatic prostate diseases.

Smaller prostate glands have been found in smokers, while increased benign prostatic hyperplasia symptoms have been found in non-smokers [4,24]. Bolet MS et al.’s study shows that as the mean number of cigarettes smoked per day increases, post-void residual urine volume and the International Prostate Symptom Score (IPSS) also increase. Thus, smoking has a negative impact on LUTS, the patient’s quality of life, and sexual functions [25].

### 4.3. Pathological Insomnia

This study showed that 4.30% of the men had insomnia. Bliwise et al. reported that sleep disturbance or insomnia was often comorbid with nocturia due to the sensation of a full bladder, leading to the need for patients to get up during the night to urinate [26]. In the present study, there was no significant association between these factors.

Three-quarters of participants in a survey of adults over 18 years of age cited a need to visit the bathroom as the most frequent reason for nocturnal awakenings [27].

LUTS are known to have a negative impact on health-related quality of life, sleep, and mental health [28,29]. LUTS tend to regress in only a few cases [29], and the overall prevalence of LUTS increases with age [30]. Though the pathogenesis of LUTS is not fully understood, it is believed to be multifactorial, including neurological, vascular, and connective tissue processes [31]. In addition to interactions among the nervous, vascular, immune, and endocrine systems [32], psychological factors may also play an important role in the presentation of LUTS [33].

### 4.4. Psychosis

All episodes of psychosis in the present study (2.86%), outside major depressive disorder, were due to paranoid schizophrenia. Leucht S et al. reported that physical illness and schizophrenia have suggested that people with schizophrenia have higher than expected rates of certain physical comorbidities [34], in addition to lower than expected rates of other physical illnesses [35]. A number of commonly reported medical comorbidities in schizophrenia include cardiovascular, neurological, genitourinary, respiratory, and gastrointestinal disorders [36].

### 4.5. Alcoholism

In the present study, alcoholism was the most significant disorder in patients with symptomatic benign prostatic disease. Alcoholism had a prevalence of 2.15% in the general sample. Thorpe et al. concluded that alcoholism has an influence on androgen metabolism, increases serum estrogen levels, and may, therefore, affect the risk of benign prostatic disease [37].

Alcoholism has been reported to reduce the risk of BPH, primarily in thinner men (BMI under 26) [38]. Similarly, diagnosis and surgery for benign prostatic disease have been shown to decrease in men with moderate consumption of alcohol (defined as 1–3 drinks per day) compared to non-drinkers [39]. However, heavy alcohol consumption (defined as self-reported alcoholism, >72 g/day [>5.1 drinks per day] or >40 g/day [>2.9 drinks per day]) had a negative effect, with increased incontinence and obstructive or irritating LUTS [40]. Nevertheless, other studies have found that heavy drinking (>40 g/day) was not a risk factor for exacerbating LUTS [41]. A meta-analysis reported that alcohol consumption was not a significant risk factor for LUTS [42]. Moderate alcohol intake has been linked with a reduced risk of coronary artery disease, and this beneficial effect on the cardiovascular system could also have had a protective effect on the progression of BPH [43].

The present study has found a clear relationship between benign prostatic disease and mental disorders, smoking, or alcoholism, mostly anxiety and depressive disorder. This, therefore, is in line with previous studies and has clear implications for approaching and treating patients, since the interrelationship of disorders and comorbidities as concomitant diseases should have implications for treatments to improve outcomes.

The diagnoses of mental disorders are susceptible to disagreement between physicians, and a significant percentage of the interviews are blindly rated by independent assessors. The authors highly considered including in further analysis a systematic retrospective review by more than one independent assessor of the hospital records and mental health diagnosis.

### 4.6. Limitations of the Study

The present study emerged as a substudy investigation within a broader cohort of 2240 men from the community. These individuals had previously taken part in a prostate health research study designed for the general population with the primary objective of identifying a diagnostic marker for prostate cancer (CH10 Code RTC-2017-6271-1, RETOS). It is important to note that the sample of men utilized for this current study was not originally intended to be the primary focus. Nevertheless, due to the way in which patients were recruited through spontaneous requests by men from the community, it has proven to be a suitable and valuable resource for the purposes of this specific investigation.

## 5. Conclusions

Unresolved symptomatic benign prostate disease is associated with a higher incidence of anxiety, depressive disorder, and psychosis compared to individuals without benign prostate disease.

Smoking is the disorder with the best prognosis, in terms of quitting smoking, in men with benign prostate disease.

Alcoholism is the disorder associated with the worst prognosis in patients with symptomatic benign prostatic disease, both when the urinary symptoms disappear, as in the case of self-limiting episodes of urinary tract infection (not prostatitis) or prostatic intraepithelial neoplasia (PIN), and when symptomatic benign prostatic disorders persist, showing continued symptoms and exacerbations such as prostatitis.

It is desirable to include an analysis of marital status, family size, occupations, and household incomes in future studies to confirm the findings.

## Figures and Tables

**Table 1:** Comparison of the distribution of concomitant diseases between GP and GU groups.

Diagnoses		GP *n* = 206		GU *n* = 352		Total *n* = 558		*p* *
		*n*	%	*n*	%	*n*	%	
Primary urological diseases	Nonbacterial prostatitis	68	33.01	108	30.68	176.00	31.54	0.5725
	Acute bacterial prostatitis	50	24.27	110	31.25	160.00	28.67	0.0817
	UTI	56	27.18	80	22.73	136.00	24.37	0.2613
	PIN	28	13.59	50	14.20	78.00	13.98	0.8997
	Prostatodynia	4	1.94	4	1.14	8.00	1.43	0.4754
Secondary urological diseases (SUDs)	None	98	47.57	170	48.30	268.00	48.03	0.9301
	HBP	72	34.95	130	36.93	202.00	36.20	0.6494
	Nocturia	0	0	2	0.57	2.00	0.36	0.5334
	Prostatism	2	0.97	6	1.70	8.00	1.43	0.7165
	Cystitis	2	0.97	6	1.70	8.00	1.43	0.7165
	UTI	14	6.80	22	6.25	36.00	6.45	0.8588
	Prostatitis	4	1.94	4	1.14	8.00	1.43	0.4754
	Chronic prostatitis	4	1.94	8	2.27	12.00	2.15	1
	UI	4	1.94	0	0	4.00	0.72	0.0182
	Prostatodynia	2	0.97	4	1.14	6.00	1.08	1
	Candidal balanitis	4	1.94	0	0	4.00	0.72	0.0182
Primary mental disorder, smoking, or alcoholism	None	0	0	290	82.39	290.00	51.97	0.0001
	Anxiety	84	40.78	26	7.39	110.00	19.71	0.0001
	Depression	24	11.65	8	2.27	32.00	5.73	0.0001
	Insomnia	18	8.74	6	1.70	24.00	4.30	0.0001
	Smoking	64	31.07	10	2.84	74.00	13.26	0.0001
	Alcoholism	2	0.97	10	2.84	12.00	2.15	0.2259
	Psychosis	14	6.80	2	0.57	16.00	2.87	0.0001
Secondary mental disorder, smoking, or alcoholism	Anxiety	2	0.97	4	1.14	6.00	1.08	1
	Depression	4	1.94	0	0	4.00	0.72	0.0182
	Insomnia	6	2.91	4	1.14	10.00	1.79	0.1839
	Smoking	30	14.56	9	2.56	39.00	6.99	0.0001
	Alcoholism	12	5.83	5	1.42	17.00	3.05	0.0048
	Psychosis	0	0	2	0.57	2.00	0.36	0.5334
	None	152	73.79	328	93.18	480.00	86.02	0.0001

GP: men who were diagnosed first in time with a mental disorder, smoking, or alcoholism. GU: men who were diagnosed first in time with a urological disease. UTI: urinary tract infection. PIN: prostatic intraepithelial neoplasia. UI: urinary incontinence. SUD: secondary urological disease. * Fisher’s exact test.

## Data Availability

The research data are available in the archive of the surgery department of the University of Salamanca and the University Hospital of Salamanca.

## Appendix A

**Table A1:** Age; BMI; PSA; PSA index; PSA velocity; mean duration of suffering from a primary mental disorder, smoking, or alcoholism (psychiatric) (months); mean duration of suffering from a primary urological disease (months); mean time between primary urological and primary mental disorder, smoking, or alcoholism (months).

Variable	Overall Sample	GA	GB	*p* *
Mean age (years)	63.3	63.13	63.40	0.7510
Mean BMI (kg/m^2^)	27.56	27.44	27.44	0.9720
Mean PSA (ng/mL)	2.83	2.66	2.94	0.1720
Mean PSA index (%)	20.15	20.27	20.08	0.3720
Mean PSA velocity across 12 months (ng/dL)	1.18	1.04	1.26	0.5600
Mean duration of suffering from a primary psychiatric disorder (months)	93.43	103.46	50.38	0.0001
Mean duration of suffering from a primary urological disease (months)	42.90	28.15	47.52	0.0001
Mean time between primary urological and psychiatric disorders (months)	75.52	79.95	56.46	0.0250

GP: men who were first diagnosed with a mental disorder, smoking, or alcoholism. GU: men who were first diagnosed with a urological disease. BMI: body mass index. PSA: prostate-specific antigen. * Fisher’s exact test.

**Table A2:** Contribution of mental disorders, smoking, or alcoholism (psychiatric) and urological disorders to each factorial axis.

General Column Points									
Psychiatric/Urological Disorder	Mass	Dimension Score		Inertia	Contribution				
		1	2		of the Point to the Inertia of the Dimension		of the Dimension to the Inertia of the Point		
					1	2	1	2	Total
Prostatitis	0.215	0.393	0.278	0.018	0.081	0.065	0.755	0.234	0.989
Acute bacterial prostatitis	0.195	0.589	0.060	0.029	0.165	0.003	0.949	0.006	0.956
UTI	0.166	0.260	−0.315	0.009	0.027	0.065	0.520	0.475	0.995
PIN	0.095	0.431	−0.452	0.013	0.043	0.076	0.579	0.395	0.974
Prostatodynia	0.010	0.030	−0.986	0.005	0.000	0.037	0.001	0.505	0.505
Anxiety	0.134	−0.757	0.864	0.057	0.187	0.393	0.552	0.446	0.999
Depression	0.039	−0.787	0.107	0.010	0.059	0.002	0.971	0.011	0.982
Insomnia	0.029	−0.847	−0.384	0.011	0.051	0.017	0.798	0.102	0.900
Smoking	0.090	−1.216	−0.915	0.074	0.325	0.296	0.739	0.260	1.000
Alcoholism	0.007	0.599	−0.724	0.005	0.006	0.015	0.223	0.202	0.426
Psychosis	0.020	−1.088	0.644	0.012	0.056	0.032	0.763	0.166	0.929
Active Total	1.000			0.243	1.000	1.000			

Correspondence analysis. UTI: urinary tract infection. PIN: prostatic intraepithelial neoplasia.
